# The Effects of Ingesting a Single Bolus of Hydrolyzed Collagen versus Free Amino Acids on Muscle Connective Protein Synthesis Rates

**DOI:** 10.1249/MSS.0000000000003788

**Published:** 2025-06-13

**Authors:** THORBEN AUSSIEKER, JEREMIAS KAISER, FLORIS K. HENDRIKS, TOM A. H. JANSSEN, JOAN M. SENDEN, JANNEAU M. X. VAN KRANENBURG, JOY P. B. GOESSENS, ANTOINE ZORENC, ESTHER KORNIPS, TJINTA BRINKHUIZEN, KEITH BAAR, TIM SNIJDERS, ANDREW M. HOLWERDA, LUC J. C. VAN LOON

**Affiliations:** 1 Department of Human Biology, Institute of Nutrition and Translational Research in Metabolism (NUTRIM), Maastricht University Medical Centre^+^, Maastricht, THE NETHERLANDS; 2 Department of Dermatology, Catharina Hospital, Eindhoven, THE NETHERLANDS; 3 Department of Dermatology, Maastricht University Medical Centre^+^, Maastricht, THE NETHERLANDS; 4 Department of Neurobiology, Physiology and Behavior, University of California Davis, Davis, CA

**Keywords:** MYOFIBRILLAR PROTEINS, CONNECTIVE TISSUE, RESISTANCE EXERCISE, GLYCINE

## Abstract

The suggested anabolic properties of hydrolyzed collagen on connective tissue may be attributed to the delivery of amino acids as precursors or to anabolic signaling properties of bioactive peptides.

**Purpose:**

This study aimed to assess the effect of ingesting a single bolus of hydrolyzed collagen or free amino acids on myofibrillar and muscle connective protein synthesis rates.

**Methods:**

In a randomized, double-blind, parallel design, 45 young male (*n* = 21) and female (*n* = 24) adults (age, 23 ± 3 yr; BMI, 22.3 ± 2.2 kg·m^−2^) received intravenous infusions with L-[*ring*-^13^C_6_]-phenylalanine. After unilateral resistance exercise, participants ingested either 30 g hydrolyzed collagen (COLL, *n* = 15), 30 g free amino acids reflecting the collagen amino acid profile (AA, *n* = 15), or a noncaloric placebo (PLA, *n* = 15). Blood and muscle tissue samples were collected over 6 h to assess myofibrillar and muscle connective protein synthesis rates and associated signaling responses.

**Results:**

Both collagen and free amino acid ingestion substantially increased circulating plasma amino acids concentrations and affected collagen turnover proteins. Collagen and free amino acid ingestion did not significantly increase myofibrillar protein synthesis rates in the rested (0.039 ± 0.011, 0.037 ± 0.010, and 0.036 ± 0.015%·h^−1^ in PLA, COLL, and AA, respectively) or the exercised (0.049 ± 0.010, 0.048 ± 0.011, and 0.045 ± 0.013%·h^−1^) leg (*P* > 0.05). Similarly, both collagen and free amino acid ingestion did not significantly increase muscle connective protein synthesis rates in the rested (0.065 ± 0.014, 0.063 ± 0.017, and 0.061 ± 0.025%·h^−1^ in PLA, COLL, and AA, respectively) or the exercised (0.098 ± 0.023, 0.092 ± 0.028, and 0.085 ± 0.024%·h^−1^) leg (*P* > 0.05).

**Conclusions:**

The ingestion of a single bolus of collagen hydrolysate or free amino acids substantially increases circulating amino acids concentrations, particularly glycine, but does not further increase myofibrillar or muscle connective protein synthesis rates at rest or during recovery from exercise in healthy, recreationally active young men and women.

Skeletal muscle is under constant remodeling, with average turnover rates of 1%–2% per day (1). Muscle tissue is made up of various proteins, with mainly myofibrillar proteins being responsible for force production and connective proteins being instrumental to ensure proper force transmission (2,3). Both myofibrillar and muscle connective protein networks are under constant remodeling to ensure that skeletal muscle tissue can adapt to the requirements imposed upon it.

Resistance exercise robustly increases muscle protein synthesis rates, resulting in increases in both muscle mass and strength (4,5). Our lab (6–10) as well as others (11–19) have shown that a single bout of resistance exercise increases both myofibrillar and muscle connective protein synthesis rates, supporting the remodeling of the contractile and connective protein network. The ingestion of protein during recovery from exercise can further augment muscle protein synthesis rates (18,20–22) and has, therefore, become a popular nutritional strategy to further improve skeletal muscle reconditioning (23,24). Interestingly, protein ingestion during recovery from resistance exercise further increases myofibrillar (8,25–28) but not muscle connective (6–8,13,15,29,30) protein synthesis rates. Consequently, there is an active debate as to whether the muscle connective protein network is responsive to protein ingestion at rest and/or during recovery from exercise.

We (8,31) as well as others (32–34) have speculated that the ingestion of dietary collagen protein may stimulate muscle connective protein synthesis rates. Dietary collagen protein ingestion does not seem to augment myofibrillar protein synthesis rates at rest or during recovery from exercise (8,35,36). This may be attributed to the amino acid composition of the collagen protein, mainly the low levels of essential amino acids and leucine in particular (32). However, the greater nonessential amino acid content of dietary collagen, specifically glycine and proline, may support improved muscle connective protein synthesis rates. Muscle connective proteins, particularly muscle collagen, is largely composed of glycine and proline. Consequently, the ingestion of hydrolyzed collagen has been hypothesized to provide ample amounts of glycine and proline that may further augment muscle connective protein synthesis rates (8,31–34). In addition, preclinical work (37–43) has suggested that hydrolyzed collagen supplements may contain bioactive peptides that act as signaling molecules to stimulate postprandial muscle connective protein synthesis rates. Despite ample speculation on the proposed anabolic properties of hydrolyzed collagen ingestion on muscle connective protein synthesis rates, there are only few studies that have assessed the effect of collagen protein ingestion on myofibrillar or muscle connective tissue protein synthesis rates at rest or during recovery from exercise (8,35,44).

In the present study, we investigated the effects of hydrolyzed collagen ingestion on both myofibrillar and muscle connective protein synthesis rates at rest and during recovery from exercise. To differentiate between the potential anabolic properties of hydrolyzed collagen ingestion on tissue protein synthesis via the provision of ample amounts of glycine and proline or via the proposed anabolic properties of potential bioactive peptides present in hydrolyzed collagen, we provided subjects with either hydrolyzed collagen or an equivalent amount of its free amino acids. In a randomized, double-blind, parallel study design, 45 healthy, young men and women ingested 30 g hydrolyzed collagen, 30 g free amino acids, or a noncaloric placebo after a single bout of unilateral resistance exercise. Primed, continuous intravenous L-[*ring*-^13^C_6_]-phenylalanine infusions were applied with blood, muscle, and even skin tissue samples being collected to assess myofibrillar, muscle connective, and skin protein synthesis rates in healthy, young, recreationally active men and women. The primary objective was to assess the effect of the ingestion of hydrolyzed collagen versus free amino acid on postexercise muscle connective protein synthesis rates *in vivo* in humans. Secondary objectives were to assess the effect of hydrolyzed collagen versus free amino acid ingestion on postexercise myofibrillar protein synthesis rates, on myofibrillar and muscle connective protein synthesis rates at rest, on skin protein synthesis rates, and to characterize plasma amino acid availability after the ingestion of hydrolyzed collagen versus free amino acids.

## METHODS

### Participants

A total of 45 healthy, recreationally active men and women (age, 23 ± 3 yr; BMI, 22.3 ± 2.1 kg·m^−2^) volunteered to participate in this parallel-group, double-blind, randomized controlled trial. Participants’ characteristics are presented in Table 1. After pretesting, participants were randomly assigned to one of three groups consuming either 30 g hydrolyzed collagen (COLL, *n* = 15), 30 g free amino acids with the same amino acid profile of the hydrolyzed collagen (AA, *n* = 15), or a noncaloric placebo (PLA, *n* = 15). All participants were informed of the nature and possible risks of the experimental procedures before their written informed consent was obtained. This study was approved by the Medical Ethical Committee of the Academic hospital Maastricht/Maastricht University and conforms to the principles outlined in the Declaration of Helsinki for use of human participants and tissue. The trial was registered at the Dutch Trial Register (NL9235) and was conducted between December 2021 and October 2022 at Maastricht University, Maastricht, The Netherlands. Clinical Trial Center Maastricht independently monitored the study. One subject in AA dropped out after feeling unwell after the exercise session. We were unable to collect muscle biopsy samples in one subject at *t* = 360 min, which resulted in exclusion of this subject in all muscle analysis. Additionally, we were unable to collect muscle biopsy samples in one subject at *t* = 180 min, which resulted in exclusion of this subject in the early vs late response muscle analysis.

**TABLE 1:** Participants’ characteristics and habitual dietary intake.

	PLA (*n* = 15)	COLL (*n* = 15)	FAA (*n* = 14)
Age (yr)	23 ± 3	23 ± 4	23 ± 4
Sex (m/f)	7/8	7/8	7/7
Height (m)	1.74 ± 0.09	1.73 ± 0.09	1.76 ± 0.09
Weight (kg)	66.7 ± 10.9	67.3 ± 13.2	70.0 ± 9.7
BMI (kg·m^−2^)	22.0 ± 1.9	22.3 ± 2.5	22.6 ± 2.4
Lean body mass (kg)	47.2 ± 8.7	48.7 ± 11.5	50.8 ± 9.8
Body fat (%)	26.4 ± 5.9	25.0 ± 8.0	27.3 ± 6.5
1RM leg press (kg)	80 ± 20	85 ± 34	83 ± 24
1RM leg extension (kg)	44 ± 11	43 ± 14	43 ± 15
Energy (MJ·d^−1^)	7.88 ± 1.97	8.05 ± 1.81	7.94 ± 2.16
Carbohydrate (g)	213 ± 67	214 ± 33	226 ± 52
Fat (g)	71 ± 23	76 ± 27	69 ± 29
Protein (g)	73 ± 21	81 ± 32	78 ± 34
Protein (g·kg^−1^·d^−1^)	1.6 ± 0.6	1.7 ± 0.5	1.5 ± 0.5
Vitamin C (mg·d^−1^)	149 ± 41	155 ± 69	155 ± 56

Values represent mean ± SD. Data were analyzed with a one-way ANOVA. There were no differences between treatments.

PLA, noncaloric flavored water; COLL, 30 g hydrolyzed collagen; FAA, 30 g free amino acid mixture; BMI, body mass index.

### Pretesting

Participants (18–35 yr old, with a BMI > 18.5 and <30.0 kg·m^−2^) underwent an initial screening session to assess height, body weight, and body composition via DXA (Hologic, Discovery A; QDR Series, Marlborough, MA). The system’s software package Apex version 4.0.2 was used to determine whole-body and segmental lean mass, fat mass, and bone mineral content. Afterward, one-repetition maximum (1RM) on the leg press and extension machines (Technogym, Rotterdam, the Netherlands) of both legs were assessed. Participants were deemed healthy based on their responses to a medical questionnaire and were excluded from participation when smoking, using medication that affected protein metabolism, having any musculoskeletal diseases, or being intolerant to the investigated protein products. The pretesting and experimental trials were separated by at least 5 d.

### Diet and physical activity

All participants refrained from strenuous physical activity and alcohol consumption and filled out food intake and physical activity questionnaires for 2 d prior to the experimental trial. Habitual dietary intake data were analyzed using online software available from the Dutch Health Council *(Mijn Eetmeter*, https://mijn.voedingscentrum.nl/nl/eetmeter/) and are presented in Table 1. Participants consumed the same standardized meal before 2100 h on the evening before the experimental trial. This prepackaged standardized meal provided 1.71 MJ, with 55% of the energy from carbohydrates, 30% energy from fat, and 15% energy from protein. In addition, participants consumed 200 mL orange juice providing 0.38 MJ and 80 mg vitamin C. Thereafter, participants remained fasted until the experimental test day.

### Study design

Participants performed a unilateral leg resistance exercise session before consuming a randomly assigned beverage (300 mL) containing 30 g hydrolyzed collagen (COLL), 30 g free amino acids (AA), or placebo (PLA). The hydrolyzed collagen (Tessenderlo Innovation Center, part of Tessenderlo Group NV, Belgium) and the free amino acids (Sigma-Aldrich, St. Louis, MO; Merck KGaA, Darmstadt, Germany; Zhangjiagang Specom Biochemical, Jiangsu, China) were dissolved in water after which beverages were flavored with vanilla flavoring (Dr. Oetker, Amersfoort, The Netherlands). The free amino acid mix provided an equivalent amount of all amino acids within the hydrolyzed collagen, with the exception of hydroxylysine (0.45 g in the hydrolyzed collagen) as it was not available in food or pharma grade quality. The noncaloric placebo was vanilla-flavored water. Amino acid profiles of the study beverages can be found in Supplemental Table 1 (Supplemental Digital Content, http://links.lww.com/MSS/D258). Randomization was performed using a computerized list randomizer (http://www.randomization.com/). Participants were sequentially allocated to the treatment groups, stratified by sex, by an independent researcher, according to the randomized list. There was an equal distribution of males and females for each group (seven males and eight females per condition). The study beverages were prepared by an independent researcher and provided in nontransparent plastic containers. All women were tested in the same phase of the menstrual cycle (within the first 7 d of the start of the menses, representing the follicular phase).

### Experimental protocol

For a schematic representation of the primed continuous infusion protocol, see Supplemental Figure 1 (Supplemental Digital Content, http://links.lww.com/MSS/D258). At ∼07:45 am, participants arrived at the laboratory in an overnight fasted state. A catheter was inserted into an antecubital vein for stable isotope amino acid infusion. Subsequently, a second catheter was inserted into a dorsal hand vein of the contralateral arm for arterialized venous blood sampling. To obtain arterialized blood samples, the hand was placed in a hot box (60°C) for 10 min before blood sample collection (45). After taking a baseline blood sample (*t* = −240 min), the plasma phenylalanine pool was primed with a single intravenous dose (priming dose) of L-[*ring*-^13^C_6_]-phenylalanine (3.15 μmol · kg^−1^) and L-[3,5-^2^H_2_]-tyrosine (1.20 μmol · kg^−1^). After priming, continuous intravenous infusion of L-[*ring*-^13^C_6_]-phenylalanine (0.070 μmol · kg^−1^ · min^−1^) and L-[3,5-^2^H_2_]-tyrosine (0.027 μmol · kg^−1^ · min^−1^) was initiated and maintained using a calibrated pump (Space plus infusomat; Braun, Melsungen, Germany). After resting in a supine position for an hour (*t* = −180 min), an arterialized blood sample was obtained, and a muscle biopsy sample was collected from the *vastus lateralis* muscle. While maintaining a supine position, additional arterialized blood samples were drawn (*t* = −120 min; *t* = −90 min; *t* = −60 min). After resting for another 15 min (*t* = −45 min), participants initiated the unilateral resistance exercise session (described below). Immediately after the exercise intervention (*t* = 0 min), an arterialized blood sample was obtained, and a muscle biopsy sample was collected from the *vastus lateralis* muscle of the nonexercised leg. Subsequently, participants received a 300-mL beverage corresponding to their randomly assigned treatment allocation (COLL, *n* = 15; AA, *n* = 15; PLA, *n* = 15). Immediately after the ingestion of the beverage, a skin biopsy from the buttock region of the nonexercised leg was taken (*t* = 0 min). Sequential arterialized blood samples were collected at *t* = 15, 30, 45, 60, 90, and 120 min throughout the postprandial period. At *t* = 180 min, an arterialized blood sample was obtained, and a muscle biopsy sample was collected from the *vastus lateralis* muscle of both the exercised and the nonexercise legs to determine early phase postprandial myofibrillar and muscle connective protein synthesis rates at rest and during recovery from exercise (*t* = 0–180 min). Additional arterialized blood samples were collected at *t* = 240 and 300 min. At *t* = 360 min, an arterialized blood sample was obtained, and muscle biopsy samples were collected from the *vastus lateralis* muscle of both legs to determine later phase and full postprandial myofibrillar and muscle connective protein synthesis rates (*t* = 180–360 min; *t* = 0–360 min). Afterward, a second skin biopsy from the buttock region of the nonexercised leg was taken (*t* = 360 min). When the experimental protocol was complete, the cannulas were removed, and participants consumed a light meal and were monitored for ∼30 min before leaving the laboratory.

### Blood, muscle, and skin tissue sampling

Blood samples were collected into EDTA-containing tubes and centrifuged at 1000*g* for 15 min at 4°C. Aliquots of plasma were frozen in liquid nitrogen and stored at −80°C. Muscle biopsy samples were collected using a 5-mm Bergström needle custom adapted for manual suction. Samples were obtained from separate incisions from the middle region of the *vastus lateralis*, ∼15 cm above the patella and ∼3 cm below entry through the fascia, under 1% xylocaine local anesthesia with adrenaline (1:100,000). Muscle samples were freed from any visible nonmuscle material, immediately frozen in liquid nitrogen, and stored at −80°C until further processing. Skin biopsies samples were obtained from the buttock region (above the area between the posterior and the anterior gluteal line) by the punch biopsy technique as described by Zuber (46) using a 4-mm punch biopsy instrument (KAI Medical, Solingen, Germany) under 1% xylocaine local anesthesia with adrenaline (1:100,000). The skin was penetrated by the skin punch biopsy instrument until the subcutaneous fat was reached. The skin layers were lifted, and the skin was cut free from subcutaneous tissues by using scissors. Skin samples were freed from any visible nonskin material, immediately frozen in liquid nitrogen, and stored at −80°C until further processing.

### Resistance exercise session

All participants followed the same unilateral resistance exercise protocol that consisted of six sets on the leg press and leg extension machines (Technogym, Rotterdam, the Netherlands). The exercise leg was randomized. For each exercise, participants performed six sets of 8–10 repetitions. The first two sets of both exercises were performed at 55% and 65% 1RM, respectively, and sets 3–6 at 75% 1RM. Resting periods of 2 min were allowed between sets and 2 min between exercises. Rating of perceived exertion was evaluated by the Borg scale (6–20).

### Plasma analysis

Plasma glucose and insulin concentrations were analyzed using commercially available kits (GLUC3, Roche, Ref: 05168791 190, and Immunologic, Roche, Ref: 12017547 122, respectively). Quantification of plasma amino acid concentrations was performed using ultraperformance liquid chromatograph mass spectrometry (UPLC-MS; ACQUITY UPLC H-Class with QDa; Waters, Saint-Quentin, France). A total of 50 μL of blood plasma was deproteinized using 100 μL of 10% SSA with 50 μM of MSK-A2 internal standard (Cambridge Isotope Laboratories, Tewksbury, MA). Subsequently, 50 μL of ultrapure demineralized water was added, and samples were centrifuged (15 min at 21,000 *g*). After centrifugation, 10 μL of supernatant was added to 70 μL of Borate reaction buffer (Waters). In addition, 20 μL of AccQ-Tag derivatizing reagent solution (Waters) was added after which the solution was heated to 55°C for 10 min. An aliquot of 1 μL was injected and measured using UPLC-MS. Plasma L-[ring-^13^C_6_]-phenylalanine enrichments were determined by UPLC-MS. For this, plasma phenylalanine was derivatized to its 6-aminoquinolyl-N-hydroxysuccinimidyl carbamate derivative, and enrichments were determined by UPLC-MS by using mass detection of masses 336, 342, and 346 for unlabeled and (^13^C_6_ and^13^C_9_-^15^N)-labeled phenylalanine, respectively. We applied standard calibration curves in all isotopic enrichment analyses to assess the linearity of the mass spectrometer and to control for the loss of tracer.

### Muscle tissue analysis

Muscle connective and myofibrillar protein–enriched fractions were isolated from ~100 mg wet muscle tissue by hand homogenizing on ice using a pestle in a standard extraction buffer (10 μL·mg^−1^). The samples were spun for 15 min at 700*g* and 4°C. The supernatant was transferred to a separate tube for Western blot analysis. The pellet was washed with 400 μL of extraction buffer before vortexing and centrifugation at 700*g* and 4°C for 10 min. The supernatant was removed, and the pellet was washed with 500 μL ddH_2_O before vortexing and centrifugation at 700*g* and 4°C for 10 min. The supernatant was removed, 1 mL of homogenization buffer was added, and the material was suspended by vortexing before transferring into microtubes containing 1.4 mm ceramic beads and Lysing Matrix D (MP Biomedicals, Irvine, CA). The microtubes were vigorously shaken four times for 45 s at 5.5 m·s^−1^ (FastPrep-24 5G, MP Biomedicals) to mechanically lyse the protein network. Samples were then left to rest at 4°C for 3 h before centrifugation at 700*g* and 4°C for 20 min, discarding the supernatant and adding 1 mL of homogenization buffer. The microtubes were shaken for 40 s at 5.5 m·s^−1^ before centrifugation at 700*g* and 4°C for 20 min. The supernatant was discarded, and 1 mL of KCl buffer was added to the pellets before being vortexed and left to rest overnight at 4°C. The next morning, samples were vortexed and centrifuged at 1600*g* for 20 min at 4°C where the supernatant was used for myofibrillar protein isolation and the pellet for muscle connective protein isolation.

For the myofibrillar isolation, the supernatant was transferred to a separate tube. Then 3.4 mL EtOH 100% was added, and samples were vortexed, left for 2 h at 4°C, and then centrifuged at 1600*g* for 20 min at 4°C. The supernatant was discarded, and EtOH 70% was added to the pellet, vortexed, and centrifuged again at 1600*g* for 20 min at 4°C. The supernatant was again discarded, and the remaining pellet was suspended in 2 mL of 6 M HCl in glass screw-cap tubes and left to hydrolyze overnight at 110°C.

For the connective protein isolation, the pellet, containing both immature and mature connective proteins, was mixed with 1 mL KCl buffer and left for 2 h at 4°C. The samples were vortexed and centrifuged at 1600*g* for 20 min at 4°C, and the supernatant was discarded. To the pellet, 1 mL ddH_2_O was added, vortexed, left for 2 h at 4°C, and then centrifuged at 1600*g* for 20 min at 4°C. The supernatant was removed, and the remaining pellet was suspended in 1 mL of 6 M HCl in glass screw-cap tubes and left to hydrolyze overnight at 110°C.

After hydrolyzation, the free amino acids were dissolved in 25% acetic acid solution, passed over cation exchange AG 50 W-X8 resin columns (mesh size, 100–200; ionic form, hydrogen; Bio-Rad Laboratories, Hercules, CA), washed five times with water, and finally eluted with 2 M NH_4_OH. To determine myofibrillar and connective protein L-[*ring*-^13^C_6_]-phenylalanine enrichments by GC-IRMS analysis, the purified amino acids were converted into *N*-ethoxycarbonyl ethyl ester derivatives with ethyl chloroformate. The samples were measured using a gas chromatography–isotope ratio mass spectrometer (Finnigan MAT 252; Thermo Fisher Scientific, Bremen, Germany) equipped with an Ultra I GC-column (no. 19091A-112; Hewlett-Packard, Palo Alto, CA) and combustion interface II (GC-C-IRMS). Ion masses 44, 45, and 46 were monitored for ^13^C phenylalanine. By establishing the relationship between the enrichment of a series of L-[*ring*-^13^C_6_]-phenylalanine, the standards of variable enrichment, and the enrichment of the N(O,S)-ethoxycarbonyl ethyl esters of these standards, the muscle protein–bound enrichment of phenylalanine was determined.

### Western blotting

As described in the Muscle tissue analysis section, the supernatant from the first step of the muscle fraction isolation protocol was used for Western blot analyses and was homogenized in seven volumes Tris buffer (20 mM Tris–HCL, 5 mM EDTA, 10 mM Na-pyrosphosphate, 100 mM NaF, 2 mM Na_3_VO_4_, 1% Nonident P-40; pH 7.4) supplemented with protease and phosphatase inhibitors: aprotinin 10 μg mL^−1^, leupeptin 10 μg mL^−1^, benzamidin 3 mM, and phenylmethylsulphonyl fluoride 1 mM. After homogenization, each muscle extract was centrifuged for 10 min at 10 000*g* (4°C), followed by a Bradford assay. Sample buffer was added to the supernatant to final concentrations of 60 mm Tris, 10% glycerol, 20 mg mL^−1^ SDS, 0.1 mm dithiothreitol, and 20 μg mL^−1^ bromophenol blue. The supernatant was then heated for 5 min at 100°C and immediately placed on ice. Immediately before analysis, the muscle extraction sample was warmed to 50°C and centrifuged for 1 min at 1000 *g*. The total amount of sample loaded on the gel was based on protein content. Fifty micrograms of protein was loaded in each lane. Samples were run on a Criterion Precast TGX 4%–15% gel (order no. 567-8085; Bio-Rad) ± 90 min at 150 V (constant voltage) and transferred onto a Trans-blot Turbo 0.2 μm nitrocellulose membrane (order no. 170-4159; Bio-Rad) in 10 min at 2.5 A and 25 V. Specific proteins were detected by overnight incubation at 4°C on a shaker with specific antibodies in 50% in PBS Odyssey blocking buffer (part no. 927-40 000; Li-Cor Biosciences, Lincoln, NE) after blocking for 60 min at RT in 50% in PBS Odyssey blocking buffer. Primary antibodies used were as follows: Pro-Collagen SP1.D8 (Developmental Studies Hybridoma Bank, Iowa City, IA, 1:500), phosphor-eEF2 (Thr 56) (no. 2331, 1:1000), phosphor-ribosomal S6 (Ser 240/244) (no. 5364, 1:3000), phosphor-P44/42 MAPK (ERK 1/2) (Thr 202/Tyr 204) (no. 4370, Cell Signaling Danvers, MS, 1:1500), MMP8 (sc-514803, 1:500), and MMP1 (sc-58377; Santa Cruz Biotechnology, Dallas, TX, 1:2500). After incubation, membranes were washed three times 10 min in 0.1% PBS–Tween 20 and once for 10 min in PBS. Next, samples were incubated on a shaker (1 h at RT) with infrared secondary antibodies 1:10.000 for 1 h at room temperature. Secondary antibodies used were as follows: Li-Cor IRDye 800CW Goat anti Rabbit IgG (926-32211) or Li-Cor IRDye 800CW Goat anti Mouse IgG (926-32210). After a final wash step (3 × 10 min) in 0.1% Tween 20–PBS and once 10 min in PBS, protein quantification was performed by scanning on an Odyssey Infrared Imaging System (Li-Cor Biosciences). Stain-free gel staining method was used to standardize for the amount of protein loaded. Phosphorylation status as a proxy of activation of the signaling proteins was expressed relative to the total amount of each protein.

### Skin tissue analysis

Skin protein–enriched fractions were isolated from 30 to 50 mg wet skin tissue. First, samples were crunched using an ice-cold pestle and mortar. Samples were then homogenized in 1 mL ice-cold 2% perchloric acid (PCA) using a sonic dismembrator (model 120; Fisher Scientific, Hampton, New Hampshire) for 4 times 15 s at 60% intensity. Samples were then left on ice for 10 min before centrifugation at 1000*g* and 4°C for 20 min. Afterward, the supernatant was removed for free amino acid enrichment measurements. The pellet was washed three times with 1 mL ice-cold 2% PCA before vortexing and centrifugation at 1000*g* and 4°C for 10 min, after which the supernatant was removed. After the third wash, the remaining pellet was suspended in 3 mL of 6 M HCl in glass screw-cap tubes and left to hydrolyze for 18 h at 110°C. After hydrolyzation, the samples were processed as described above in the last part of the Muscle tissue analysis section.

### Whole-body collagen turnover markers

Procollagen type I N propeptide (P1NP) and carboxy-terminal crosslinking telopeptide of type I collagen (CTX-I) were selected as markers of bone/collagen formation and bone/collagen resorption, respectively, in line with the recommendation by the International Osteoporosis Foundation and the International Federation of Clinical Chemistry (47). P1NP and CTX-I were measured at four time points from plasma samples (*t* = 0; *t* = 60; *t* = 180; *t* = 360 min). Intact PINP and CTX-I were measured using chemiluminescent immunometric assays on the IDS-iSYS instrument (Immunodiagnostic Systems, PLC) by the Central Diagnostic Laboratory at the Maastricht University Medical Centre (The Netherlands).

### Calculations

The fractional synthetic rates (FSR) of myofibrillar, muscle connective protein, and skin protein were calculated by dividing the increment in myofibrillar, muscle connective protein, and skin protein enrichment by weighted mean precursor (plasma) amino acid tracer enrichment. Consequently, myofibrillar, muscle connective, and skin protein FSR were calculated as follows:


$$\mathrm{FSR}\;\left(\%\cdot h^{-1}\right)=\left(\frac{E_{m2}-E_{m1}}{E_{\text{precursor}}\times t}\right)\times 100.\;\left[1\right]$$

*E*_m1_ and *E*_m2_ represent protein-bound L-[*ring*-^13^C_6_]-phenylalanine, *E*_precursor_ represents the average plasma free L-[*ring*-^13^C_6_]-phenylalanine enrichment during the tracer incorporation period, and *t* indicates the time interval (h) between biopsies.

### Statistical analysis

An *a priori* sample size calculation was performed with differences in postprandial muscle connective protein synthesis rates between the groups as primary outcome measure. A minimum sample size of 14 participants per treatment was calculated using a power of 80%, a significance level of 0.01667 (adjusted from 0.05 for multiple comparisons), an SD of 0.016%·h^−1^, and a difference in muscle connective protein synthesis rates of 0.021%·h^−1^ between treatments (or ~20% when expressed as a relative difference). To account for potential dropouts, we included a total of 15 participants per group. The normality of the data was checked using the Shapiro–Wilk test. Sphericity was checked using Mauchly’s test of sphericity, and in case that test was significant, the Greenhouse–Geisser corrected values were used for interpretation of the repeated-measures ANOVA. Baseline characteristics and dietary intake between groups were compared using a one-way ANOVA. The trapezoidal rule adjusted to baseline concentration (*t* = 0 min) was applied to calculate the incremental area under curve (iAUC) of the amino acid concentrations. Time-dependent variables (i.e., plasma glucose, insulin, amino acid concentrations, tracer enrichments, and plasma collagen turnover markers) were analyzed by a mixed-model ANOVA with time as a within-participants factor and treatment group as a between-participants factor. The analysis was conducted for the period starting at the time of hydrolyzed collagen, free amino acid, or placebo ingestion (*t* = 0 min) until the end of the experimental trial (*t* = 360 min). In case of a significant interaction effect, individual time points were analyzed using a one-way ANOVA with the time points as the dependent variable and treatment as the independent variable. Myofibrillar and connective protein FSR were analyzed by a repeated-measures ANOVA, with condition (basal, exercised, and rested) as a within-participants factor and treatment group as a between-participants factor. A secondary statistical analysis was performed on myofibrillar and muscle connective protein FSR in a time-dependent manner with early and late postprandial FSR using a three-factor repeated-measure ANOVA, with time and condition as a within-participants factor and treatment group as a between-participants factor. Protein signaling data were analyzed by a repeated-measures ANOVA with condition (basal, exercised at *t* = 180 and 360 min, rested at *t* = 180 and 360 min) as a within-participants factor and treatment group as a between-participants factor. Non–time-dependent variables (i.e., skin FSR and iAUC) were compared between treatment groups using a one-way ANOVA. Bonferroni-corrected *post hoc* comparisons were performed where appropriate. Statistical significance was set at *P* < 0.05. All data in text and figures are expressed as mean ± SD. All calculations were performed using SPSS 28.0 (SPSS Inc., Chicago, IL).

## RESULTS

### Participants’ characteristics and habitual dietary intake

There were no significant differences in participant characteristics between the three treatment groups (Table 1). Similarly, there were no differences in habitual dietary intake between treatment groups (Table 1). Dietary vitamin C intake in the 2 d before the experimental test day averaged 149 ± 41, 155 ± 69, and 155 ± 56 mg·d^−1^ in PLA, COLL, and AA, respectively (*P* = 0.95). Therefore, all participants ingested amounts of vitamin C above the recommended dietary allowance of 90 mg·d^−1^ for men and 75 mg·d^−1^ for women in the days preceding the experimental protocol day (48).

### Plasma glucose and insulin concentrations

Plasma glucose concentrations declined over time in PLA (*P* < 0.05, Supplemental Fig. 2A (Supplemental Digital Content), http://links.lww.com/MSS/D258). After hydrolyzed collagen or free amino acid ingestion, plasma glucose concentrations increased at *t* = 15–30 min and then decreased toward baseline levels, with values exceeding those observed in PLA at *t* = 30–180 min in COLL and AA, at *t* = 15 and *t* = 240 min in COLL only (time–treatment group interaction *P* < 0.05; Supplemental Fig. 2A (Supplemental Digital Content), http://links.lww.com/MSS/D258). Both hydrolyzed collagen and free amino acid ingestion increased circulating insulin concentrations, with values exceeding those observed in the placebo treatment at *t* = 30–120 min (time–treatment group interaction effect *P* < 0.05; Supplemental Fig. 2B (Supplemental Digital Content), http://links.lww.com/MSS/D258).

### Plasma amino acid concentrations

**FIGURE 1:**
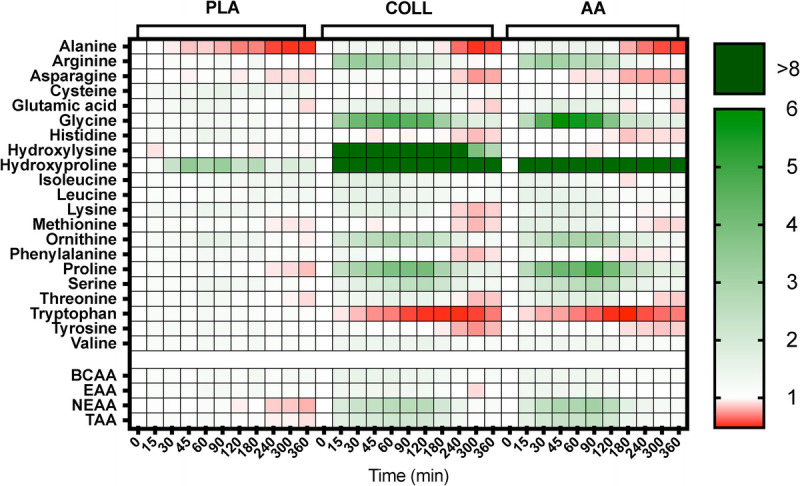
Heat map of fold changes from *t* = 0 in plasma amino acid concentrations during the experimental test day with placebo, hydrolyzed collagen, or free amino acid ingestion during recovery from a single bout of unilateral leg resistance exercise. PLA, placebo (water, *n* = 15); COLL, 30 g hydrolyzed collagen (*n* = 15); AA, 30 g free amino acid mixture (*n* = 14). TAA, total amino acids; EAA, essential amino acids; BCAA, branched-chain amino acids; NEAA, nonessential amino acids. For hydroxyproline and hydroxylysine, values under the detection limit were set to 0. Values of *t* = 0 were set to 1.

**FIGURE 2:**
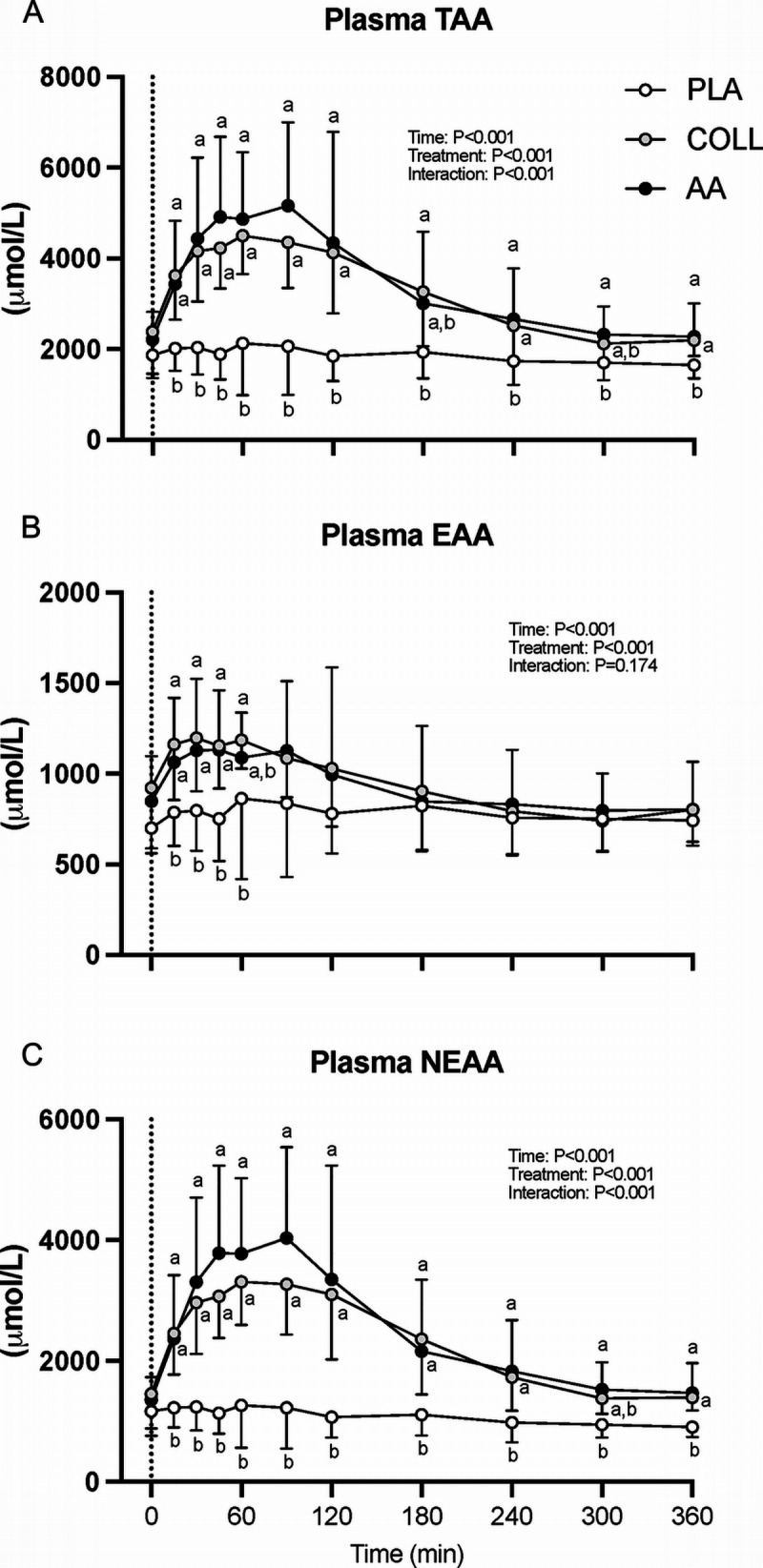
Postprandial plasma amino acid concentrations after placebo, hydrolyzed collagen, or free amino acid ingestion during recovery from a single bout of unilateral leg resistance exercise (*t* = 0–360 min). Data are displayed for TAA (A), EAA (B), and NEAA (C). The *dotted line* within the left column graphs represents the ingestion of the test drink. Values represent mean ± SD. Data were analyzed by a two-factor repeated-measures ANOVA. Bonferroni *post hoc* testing was used to detect differences between groups. Treatments without a common letter differ, *P* < 0.05. PLA, placebo (water, *n* = 15); COLL, 30 g hydrolyzed collagen (*n* = 15); AA, 30 g free amino acid mixture (*n* = 14). TAA, total amino acids; EAA, essential amino acids; NEAA, nonessential amino acids.

**FIGURE 3:**
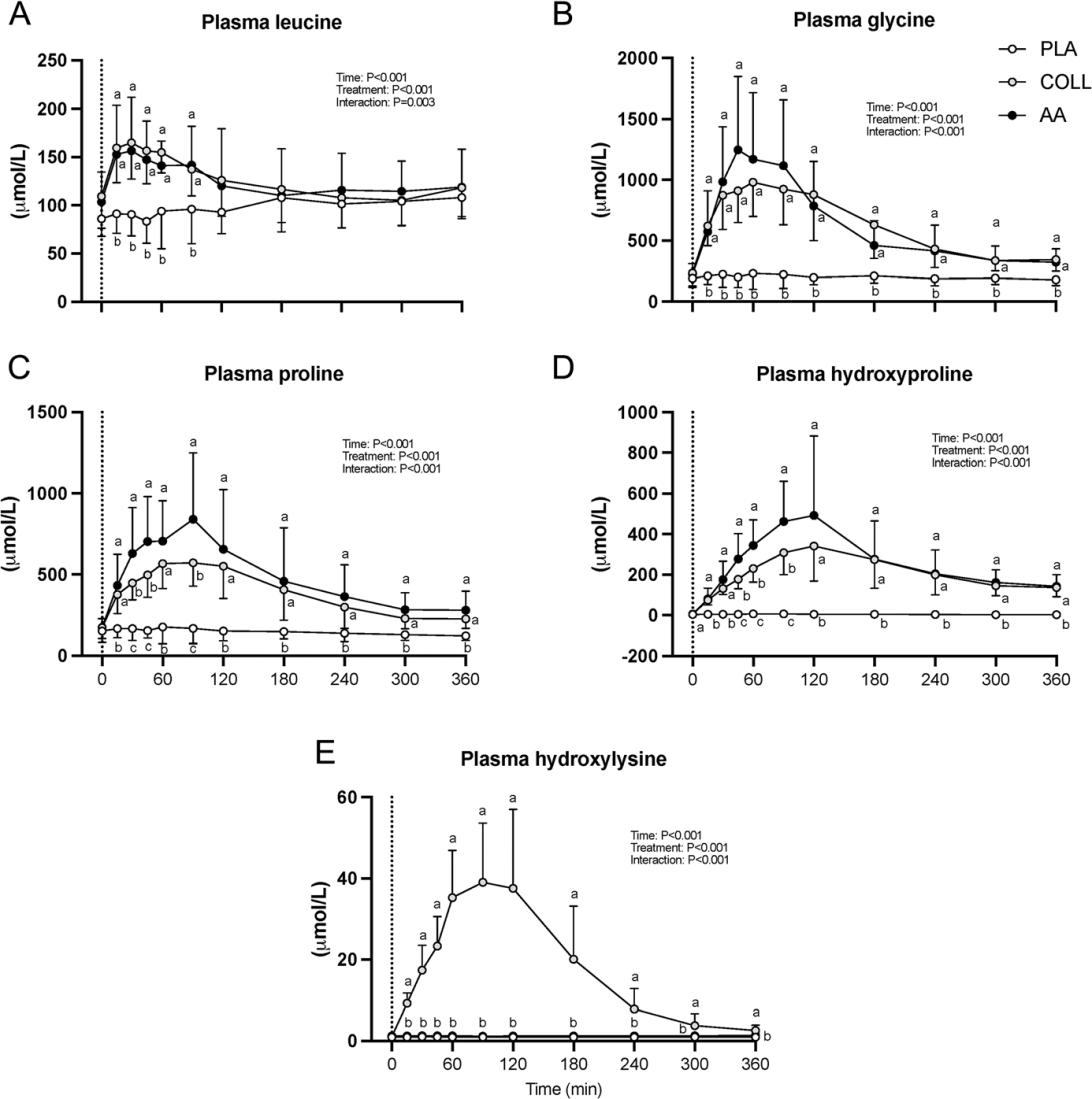
Postprandial plasma leucine, glycine, proline, hydroxyproline, and hydroxylysine concentrations after placebo, hydrolyzed collagen, or free amino acid ingestion during recovery from a single bout of unilateral leg resistance exercise (*t* = 0–360 min). Data are displayed for leucine (A), glycine (B), proline (C), hydroxyproline (D), and hydroxylysine (E). The *dotted line* represents the ingestion of the test drink. Values represent mean ± SD. Data were analyzed by a two-factor repeated-measures ANOVA. Bonferroni *post hoc* testing was used to detect differences between groups. Treatments without a common letter differ, *P* < 0.05. PLA, placebo (water, *n* = 15); COLL, 30 g hydrolyzed collagen (*n* = 15); AA, 30 g free amino acid mixture (*n* = 14).

Results for all measured amino acids are visualized in a heat map showing the fold change in plasma amino acid concentrations after hydrolyzed collagen, free amino acid, and placebo ingestion compared with baseline values, which were set at 1 (Fig. 1). Plasma amino acid concentrations are shown in Figures 2 and 3. Significant time–treatment group interactions were observed for total (TAA) and essential amino acid (EAA), leucine, glycine, proline, hydroxyproline, and hydroxylysine concentrations (all *P* < 0.001). Hydrolyzed collagen and free amino acid ingestion increased plasma total TAA, EAA, nonessential amino acid (NEAA), leucine, glycine, proline, and hydroxyproline concentrations. Free amino acid ingestion further increased plasma proline concentrations from *t* = 30 to 45 min and plasma hydroxyproline concentrations from *t* = 45 to 90 min compared with hydrolyzed collagen ingestion (*P* < 0.05). Only the ingestion of hydrolyzed collagen increased plasma hydroxylysine concentrations. The iAUC values over the whole postprandial period of plasma TAA were higher in AA compared with PLA (*P* < 0.05). There were no differences in the iAUC values over the whole postprandial period of plasma EAA and leucine between all three groups (*P* = 0.70 and *P* = 0.86, respectively). The iAUC results over the entire postprandial period of plasma NEAA, glycine, proline, and hydroxyproline were higher in COLL and AA compared with PLA (*P* < 0.05), with no differences between COLL and AA (*P* = 0.07, *P* = 0.10, *P* = 0.16 and *P* = 0.35, respectively).

### Stable isotope tracer analyses

Analysis of plasma L-[*ring*-^13^C_6_]-phenylalanine enrichments revealed a significant time–treatment group interaction effect (*P* < 0.05; Supplemental Fig. 3 (Supplemental Digital Content), http://links.lww.com/MSS/D258). At *t* = 15–90 min, plasma L-[*ring*-^13^C_6_]-phenylalanine enrichments were higher in PLA when compared with COLL and AA (*P* < 0.05). At *t* = 45–60 min, plasma L-[*ring*-^13^C_6_]-phenylalanine enrichments were the higher in AA when compared with COLL (*P* < 0.05). However, time-weighted plasma L-[*ring*-^13^C_6_]-phenylalanine enrichments over the entire 4-h basal and 6-h postprandial period did not differ between groups (*P* = 0.25 and *P* = 0.11, respectively).

### Myofibrillar protein synthesis

**FIGURE 4:**
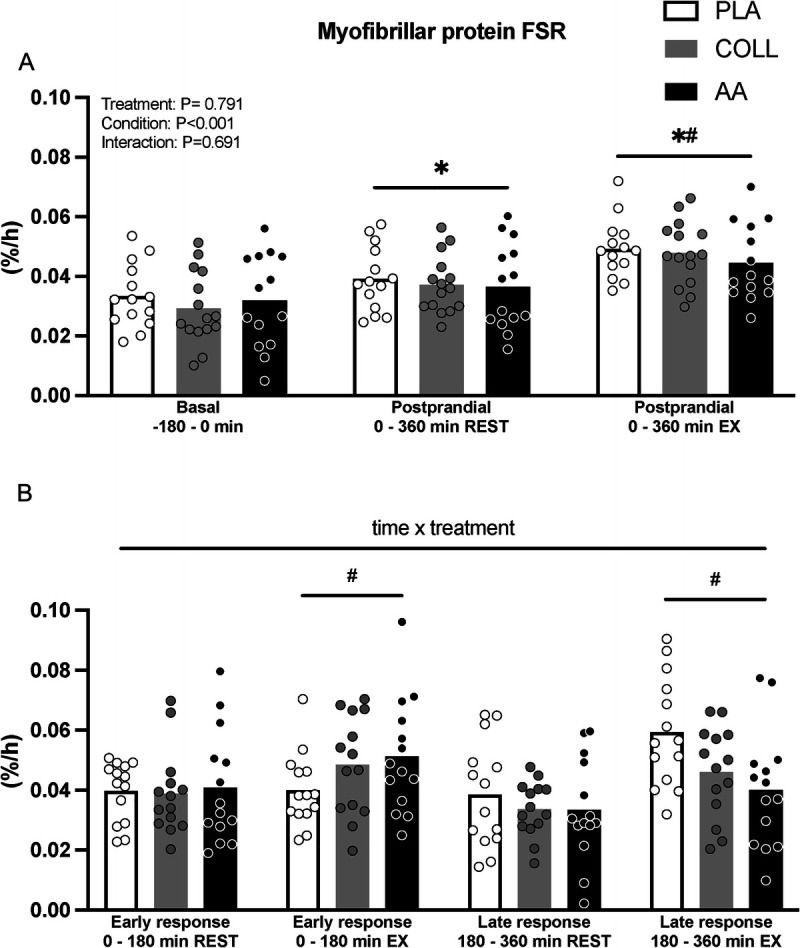
Fractional myofibrillar protein synthesis rates (%^.^h^−1^) after placebo, hydrolyzed collagen, or free amino acid ingestion during recovery from a single bout of unilateral leg resistance exercise. A. Values for basal (*t* = −240–0 min) and full postprandial rates (*t* = 0–360 min). B. Values for the early (*t* = 0–180 min) and late (*t* = 180–360 min) postprandial response. Values represent mean ± SD. Data were analyzed by a two-factor repeated-measures ANOVA (A) and a three-factor repeated-measures ANOVA (B). Bonferroni *post hoc* testing was used when appropriate. * Different from basal, *P* < 0.05. # Different from REST, *P* < 0.05. FSR, fractional synthesis rate; PLA, placebo (water, *n* = 14); COLL, 30 g hydrolyzed collagen (*n* = 15; 14 for 7B); AA, 30 g free amino acid mixture (*n* = 14); REST, rested leg: EX, exercised leg.

Postabsorptive myofibrillar protein synthesis rates averaged 0.033 ± 0.011, 0.029 ± 0.012, and 0.032 ± 0.016%·h^−1^ in the PLA, COLL, and AA groups, respectively (Fig. 4A). Postprandial myofibrillar protein synthesis rates in the rested leg over the full 6-h period were significantly higher compared with postabsorptive rates at 0.039 ± 0.011, 0.037 ± 0.010, and 0.036 ± 0.015%·h^−1^ in the PLA, COLL, and AA groups, respectively (time effect *P* < 0.05; Fig. 4A). Postprandial myofibrillar protein synthesis rates in the exercised leg over the full 6-h period were significantly higher compared with postabsorptive and rested rates at 0.049 ± 0.010, 0.048 ± 0.011, and 0.045 ± 0.013%·h^−1^ in the PLA, COLL, and AA groups, respectively (time effect *P* < 0.05; Fig. 4A). There were no differences between treatment groups for myofibrillar protein synthesis rates in the basal state and during the full 6-h postprandial period in the rested or exercised leg (*P* = 0.69, *P* = 0.79, and *P* = 0.62, respectively).

A secondary analysis for myofibrillar protein synthesis rates in the early (*t* = 0–180 min) and late (*t* = 180–360 min) recovery from exercise period was performed and revealed a significant time–treatment group interaction effect (*P* < 0.05; Fig. 4B). In both the early and the late recovery periods, the exercised leg showed higher myofibrillar protein synthesis rates when compared with the nonexercised leg (*P* < 0.05) with no differences between treatment groups (*P* = 0.72).

An additional statistical analysis, not part of the statistical analysis plan, was performed to assess potential sex-based differences in myofibrillar protein synthesis rates. Postabsorptive myofibrillar protein synthesis rates were higher in women compared with men (Supplemental Fig. 4, Supplemental Digital Content, http://links.lww.com/MSS/D258, *t*-test: *P* < 0.05). Furthermore, the absolute changes in myofibrillar protein synthesis rates from the basal state to the full 6-h postprandial period of the rested and exercised leg were calculated. Treatment groups were merged as there was no treatment effect in the primary analysis. Men showed greater postprandial increases in myofibrillar protein synthesis rates in the rested (0.011 ± 0.013 and 0.001 ± 0.011%·h^−1^ for men and women, respectively, *t*-test: *P* < 0.05) as well as the exercised (0.023 ± 0.012 and 0.009 ± 0.012%·h^−1^ for men and women, respectively, *t*-test: *P* < 0.05) leg when compared with women.

### Muscle connective protein synthesis

**FIGURE 5:**
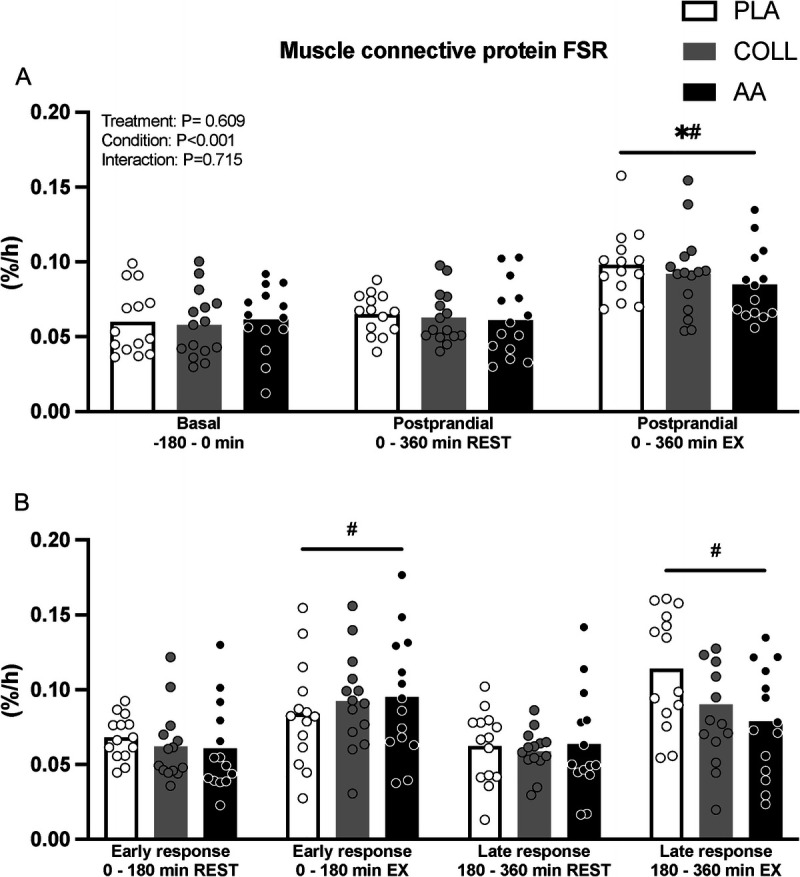
Fractional muscle connective protein synthesis rates (%^.^h^−1^) after placebo, hydrolyzed collagen, or free amino acid ingestion during recovery from a single bout of unilateral leg resistance exercise. A. Values for basal (*t* = −240–0 min) and full postprandial rates (*t* = 0–360 min). B. Values for the early (*t* = 0–180 min) and late (*t* = 180–360 min) postprandial response. Values represent mean ± SD. Data were analyzed by a two-factor repeated-measures ANOVA (A) and a three-factor repeated-measures ANOVA (B). Bonferroni *post hoc* testing was used when appropriate. * Different from basal, *P* < 0.05. # Different from REST, *P* < 0.05. FSR, fractional synthesis rate; PLA, placebo (water, *n* = 14); COLL, 30 g hydrolyzed collagen (*n* = 15; 14 for 8B); AA, 30 g free amino acid mixture (*n* = 14); REST, rested leg: EX, exercised leg.

Postabsorptive muscle connective protein synthesis rates averaged 0.060 ± 0.022, 0.058 ± 0.022, and 0.061 ± 0.022%·h^−1^ in the PLA, COLL, and AA groups, respectively (Fig. 5A). Postprandial muscle connective protein synthesis rates in the rested leg over the full 6-h period averaged 0.065 ± 0.014, 0.063 ± 0.017, and 0.061 ± 0.025%·h^−1^ in the PLA, COLL, and AA groups, respectively (Fig. 5A). Postprandial muscle connective protein synthesis rates in the exercised leg over the full 6-h period (0.098 ± 0.023, 0.092 ± 0.028, and 0.085 ± 0.024%·h^−1^ in the PLA, COLL, and AA groups, respectively) were significantly higher compared with postabsorptive and rested rates (time effect *P* < 0.05; Fig. 5A). There were no differences between treatment groups for muscle connective protein synthesis rates in a basal state and during the full 6-h postprandial period in the rested or exercised leg (*P* = 0.93, *P* = 0.85 and *P* = 0.39, respectively).

A secondary analysis for muscle connective protein synthesis rates in the early (*t* = 0–180 min) and late (*t* = 180–360 min) recovery from exercise period was performed (Fig. 5B). In both the early and the late recovery periods, the exercised leg showed higher muscle connective protein synthesis rates when compared with the nonexercised leg (*P* < 0.05) with no differences between treatment groups (*P* = 0.56).

An additional statistical analysis, not part of the statistical analysis plan, was performed to assess potential sex-based differences in muscle connective protein synthesis rates. Postabsorptive muscle connective protein synthesis rates were higher in women compared with men (Supplemental Fig. 4, Supplemental Digital Content, http://links.lww.com/MSS/D258, *t*-test: *P* < 0.05). Furthermore, the absolute changes in muscle connective protein synthesis rates from the basal state to the full 6-h postprandial period of the rested and exercised leg were calculated. Treatment groups were merged as there was no treatment effect in the primary analysis. Men showed greater postprandial increases in muscle connective protein synthesis rates in the rested (0.013 ± 0.023 and −0.006 ± 0.021%·h^−1^ for men and women, respectively, *t*-test: *P* < 0.05) as well as the exercised (0.048 ± 0.027 and 0.017 ± 0.020%·h^−1^ for men and women, respectively, *t*-test: *P* < 0.05) leg when compared with women.

### Protein signaling

**FIGURE 6:**
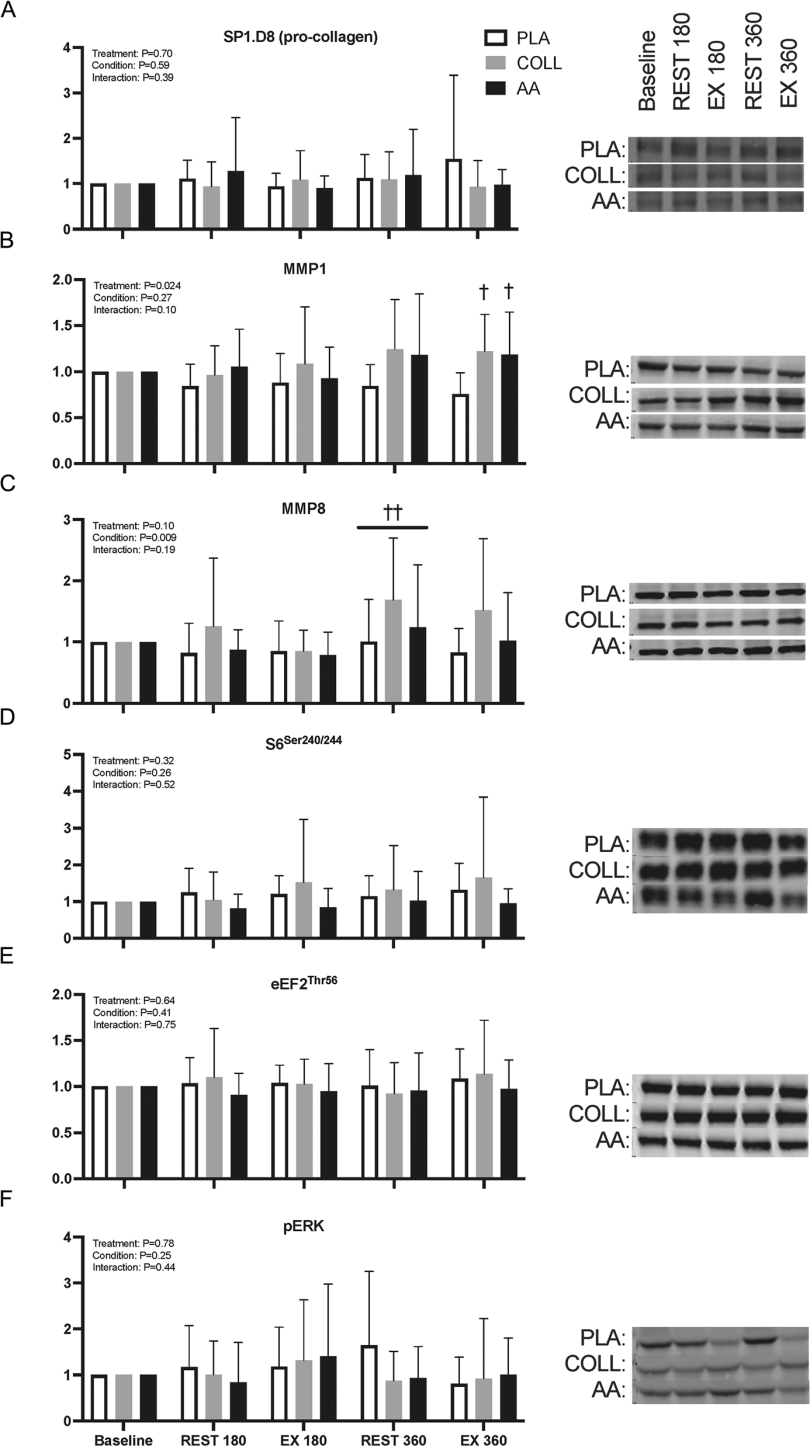
Skeletal muscle phosphorylation status (ratio of phosphorylated to total protein) of SP1.D8 (A), MMP1 (B), MMP8 (C), rpS6 (Ser240/Ser244) (D), peEF2 (Thr56) (E), and pERK (Thr 202/Tyr 204) (F), all measured by the Western blot technique. Values represent mean ± SD. Data were analyzed by a two-factor repeated-measures ANOVA. Bonferroni *post hoc* testing was used when appropriate. † Different from PLA, *P* < 0.05. †† Different from EX180, *P* < 0.05. PLA, placebo (water, *n* = 14); COLL, 30 g hydrolyzed collagen (*n* = 14); AA, 30 g free amino acid mixture (*n* = 14); REST, rested leg: EX, exercised leg.

There were no significant condition–treatment group interactions for any Western blot targets (Fig. 6, all *P* > 0.05). MMP1 level was higher in the exercised leg at *t* = 360 min for COLL and AA when compared with PLA (*P* < 0.05). MMP8 level was higher in the rested leg at *t* = 360 min when compared with the exercised leg at *t* = 180 min (*P* < 0.05), with no differences between treatment groups (*P* = 0.10).

### Skin protein synthesis

**FIGURE 7:**
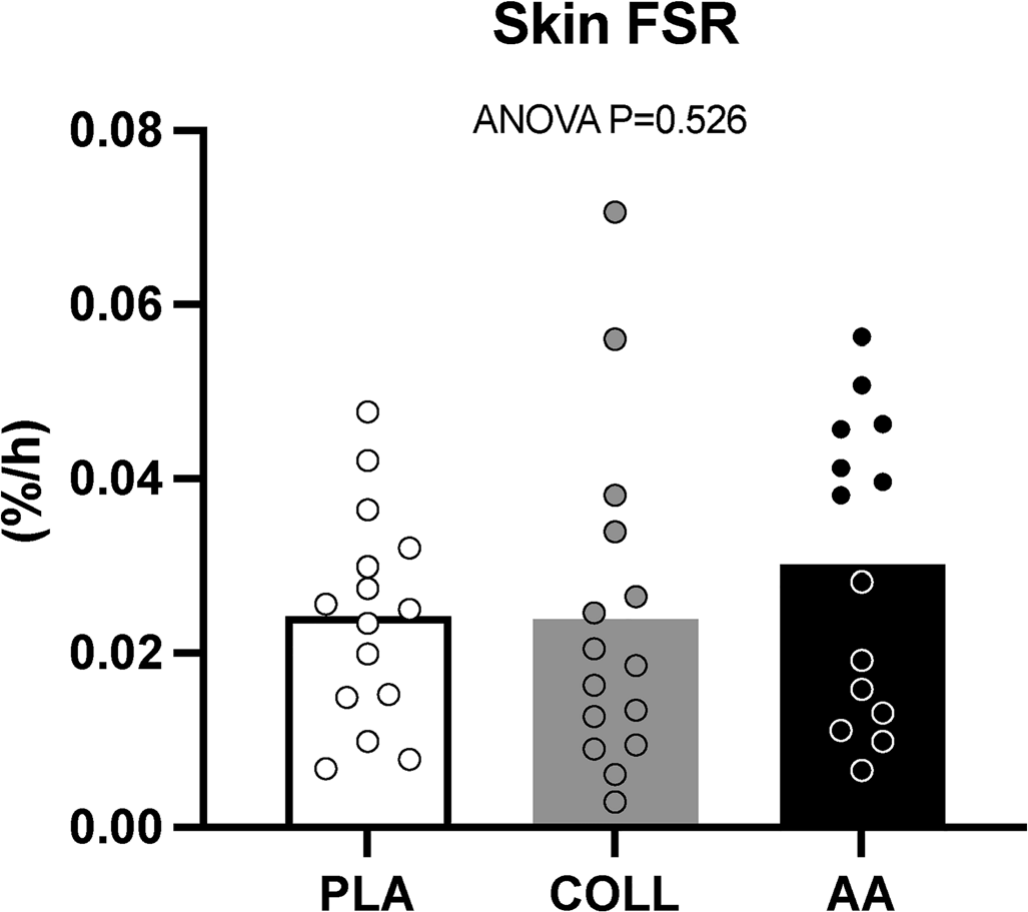
Fractional skin protein synthesis rates (%^.^h^−1^) after placebo, hydrolyzed collagen, or free amino acid ingestion during the entire postprandial period (*t* = 0–360 min) and recovery from a single bout of unilateral leg resistance exercise. Values represent mean ± SD. Data were analyzed by a one-factor ANOVA. FSR, fractional synthesis rate; PLA, placebo (water, *n* = 15); COLL, 30 g hydrolyzed collagen (*n* = 15); AA, 30 g free amino acid mixture (*n* = 14).

Skin protein synthesis rates ranged from 0.003 to 0.071%·h^−1^ and averaged 0.024 ± 0.012, 0.024 ± 0.019, and 0.030 ± 0.017%·h^−1^ in the PLA, COLL, and AA groups, respectively (Fig. 7). There were no differences in skin protein synthesis rates between treatment groups (*P* = 0.53). Skin protein synthesis rates were significantly lower when compared with myofibrillar or muscle connective protein synthesis rates (both *P* < 0.05).

### Plasma collagen turnover markers

Results for P1NP and CTX-I are shown in Supplemental Figure 5 (Supplemental Digital Content, http://links.lww.com/MSS/D258). No time–treatment group interaction was observed for plasma P1NP concentrations (*P* = 0.76). P1NP concentrations were lower at *t* = 60 min and higher at *t* = 360 min compared with the other time points (main effect of time *P* < 0.05), with no differences between treatment groups (*P* = 0.33). A significant time–treatment group interaction was observed for plasma CTX-I concentrations (*P* < 0.001), with no differences between treatment groups (*P* = 0.71). Subsequent analysis separated per group revealed no changes in CTX-I concentrations over time in PLA. In COLL, CTX-I concentrations decreased between *t* = 60–180 min and had increased beyond starting concentrations at *t* = 360 min. In AA, CTX-I concentrations decreased between *t* = 60 and 180 min and had returned to starting concentrations at *t* = 360 min.

## DISCUSSION

In the present study, an acute bout of resistance exercise substantially increased both myofibrillar and muscle connective protein synthesis rates. The ingestion of a single bolus of 30 g hydrolyzed collagen or an equivalent amount of free amino acids substantially increased circulating amino acid concentrations but did not (further) increase myofibrillar or muscle connective protein synthesis rates at rest or during recovery from exercise. In addition, we were able to measure skin protein synthesis rates, ranging between 0.003 and 0.071%·h^−1^, which were unresponsive to the ingestion of hydrolyzed collagen or the equivalent amount of free amino acids throughout a 6-h postprandial period.

The ingestion of 30 g hydrolyzed collagen rapidly increased plasma amino acid concentrations (Figs. 2 and 3). The rapid and substantial increase in plasma hydroxyproline and hydroxylysine concentrations (Fig. 3D/E) provides evidence that the ingested collagen was readily digested and absorbed, which is in line with previous findings from our lab (8) as well as others (32–34). The postprandial rise in plasma glycine and proline concentrations aligns well with previous research (8,32) and provides a rationale for the proposed efficacy of collagen supplementation to support connective tissue remodeling. Of course, the greater glycine and proline content of dietary collagen comes at the expense of other amino acids. Postprandial increases in essential amino acids after collagen ingestion (Figs. 2B) were much lower when compared with previous observations of postprandial amino acid responses after the ingestion of equivalent amounts of dairy protein (8,18,26,49). The latter may be the main reason for the inability of hydrolyzed collagen ingestion to increase myofibrillar protein synthesis rates either at rest or during recovery from exercise (8,35,36,44). Consistent with this hypothesis, we did not detect a significant further increase in myofibrillar protein synthesis rates after the ingestion of 30 g hydrolyzed collagen either at rest or during recovery from the unilateral bout of exercise when compared with the placebo group (Fig. 4). The higher myofibrillar muscle protein synthesis rates in the placebo treatment, when compared with the basal rested state, may be attributed to carry over effects of the unilateral exercise or may be secondary to a circadian effect (Fig. 4).

Despite the substantial increase in postprandial plasma glycine and proline availability, muscle connective protein synthesis rates after hydrolyzed collagen ingestion did not differ from the placebo condition (Fig. 5). These findings are in agreement with previous findings from our lab (8) as well as others (35) and provide further evidence that the acute muscle connective protein synthetic response is not affected by (hydrolyzed collagen) protein feeding. From our data, it seems evident that systemic glycine availability does not restrict the acute postprandial or postexercise increase in connective tissue protein synthesis rates (Figs. 3B and 5). Interestingly, when looking at the time response in tissue protein synthesis during recovery from exercise (Figs. 4B and 5B), there may be a time dependency with higher muscle connective protein synthesis rates after hydrolyzed collagen ingestion compared with placebo during the early as opposed to the latter stages of postexercise recovery. Although we can only speculate, this may indicate that the increase in connective tissue protein synthesis rates is augmented early, but that sufficient amino acid precursors may not be available to support synthesis over an extended period. To gain more insight into the regulation of the muscle connective protein synthetic response to amino acid precursor availability, we assessed the protein signaling levels of selected targets involved in muscle anabolism, collagen synthesis, and collagen degradation (Fig. 6). The most interesting finding was that MMP-1 and MMP-8 levels appear to show a feeding affect at 6 h, increasing in both the hydrolyzed collagen and the AA groups compared with the placebo control. We chose to look at MMP-1 and MMP-8 because they are two of the most important enzymes for degrading interstitial collagen I. Although we did not assess enzyme activity, we assessed their protein contents in muscle to evaluate potential changes over time during recovery from exercise. An increase in these enzymes could reflect the onset of greater collagen turnover. Correlation analysis revealed no relevant associations between the MMPs and also not between MMPs and muscle protein synthesis data. More work will be needed to determine whether the provision of multiple doses of hydrolyzed collagen over day(s) or weeks may augment muscle remodeling during more prolonged exercise training. For now, these findings add to the body of evidence that the ingestion of a single bolus of hydrolyzed collagen does not directly augment myofibrillar and muscle connective protein synthesis rates at rest or during acute recovery from exercise (8,35,36,44).

The proposed anabolic properties of collagen peptides have been attributed to the presence of bioactive peptides. The presence of bioactive peptides in hydrolyzed collagen has been hypothesized to function as signaling molecules that stimulate postprandial muscle connective protein synthesis rates. After the ingestion of hydrolyzed collagen, bioactive peptides have been measured in the circulation (50–52). Furthermore, *in vitro* research suggests that such peptides may stimulate connective tissue synthesis (40,42,43). To differentiate between the potential anabolic properties of hydrolyzed collagen ingestion on tissue protein synthesis via the provision of ample amounts of glycine and proline or via the proposed anabolic properties of potential bioactive peptides present in hydrolyzed collagen, we provided one group of subjects with hydrolyzed collagen and another group with an equivalent amount of the free amino acids contained in collagen. The ingestion of the free amino acid mixture substantially increased circulating plasma amino acid concentrations with levels that were similar to those observed after the ingestion of hydrolyzed collagen (Figs. 2 and 3). This further supports the previous observation that hydrolyzed collagen is rapidly digested and absorbed. In line with hydrolyzed collagen ingestion, no differences in myofibrillar or muscle connective protein synthesis rates were measured at rest or during recovery from exercise when compared with placebo (Figs. 4 and 5). Consequently, we were unable to differentiate between an acute postprandial anabolic response to either the increased provision of amino acid precursors or to the release of bioactive peptides with anabolic signaling properties.

Our data suggest that increasing plasma glycine and proline availability does not further increase muscle connective protein synthesis rates at rest or during recovery from exercise. This implies that there is little support to favor collagen over other protein sources to support muscle connective protein network conditioning during the acute stages of postexercise recovery. Collagen supplements are often proposed to support reconditioning of tissues other than muscle. It is important to note that muscle as a whole (29,53–55) and the muscle connective protein fraction contain less than 5% collagen (56). Therefore, hydrolyzed collagen ingestion may be more relevant to support recovery and reconditioning of other musculoskeletal tissues that have a greater collagen content, including ligaments, tendons, cartilage, and bone (that range from ~60% to 95% collagen) (57–60). In the present study, we did not set out to assess the effect of hydrolyzed collagen ingestion on connective tissue protein synthesis rates in these tissues. However, we did include skin biopsy sampling given its substantial collagen content (59) and the relative ease of collecting skin tissue samples (46). Skin protein synthesis rates were lower than muscle protein synthesis rates (Fig. 7 compared with Figs. 4 and 5) and are in line with previous work (29). We extend the previous work by showing that a single bolus of hydrolyzed collagen ingestion does not increase skin protein synthesis rates. Furthermore, the plasma collagen turnover marker findings revealed no differences between groups (Supplemental Fig. 4, Supplemental Digital Content, http://links.lww.com/MSS/D258). Whether (more prolonged) collagen supplementation increases connective protein synthesis rates in more collagen dense tissues, such as tendons, ligaments, cartilage, and bone, remains to be tested.

In the present study, we observed higher muscle connective when compared with myofibrillar protein synthesis rates (Figs. 4 and 5). This is in line with recent studies (15,30) but in contrast to earlier work (16,17,29). This discrepancy is unlikely to be secondary to the use of different stable isotope amino acid tracers (16,29) but rather the result of differences in the applied muscle tissue fractionation. Whereas more recent studies have applied KCl incubations to solubilize myofibrillar proteins, earlier studies (16,17,29) used an acetic acid–pepsin wash to gather an insoluble collagen pellet. The latter pellet is assumed to contain only mature collagen, which has been speculated to have a low(er) turnover rate. More work will be needed to assess potential differences in turnover of these muscle connective protein and (mature) collagen fractions. Despite differences in absolute myofibrillar and muscle connective protein synthesis rates, our study is in line with earlier studies showing an effect of exercise (16) but not of amino acid or protein provision (17,29) on muscle connective protein synthesis rates.

## CONCLUSIONS

In conclusion, an acute bout of resistance exercise increases both myofibrillar and muscle connective protein synthesis rates in recreationally active men and women. The ingestion of a single 30 g dose of hydrolyzed collagen or an equivalent amount of free amino acids increases plasma amino acid concentrations but does not increase myofibrillar or muscle connective protein synthesis rates in healthy, recreationally active, young men and women. A more sustained provision of collagen, the addition of vitamin C, longer assessment periods, and/or longer-term interventions may be required to detect the proposed benefits of collagen hydrolysate supplementation on connective tissue remodeling.
